# Combination of AAV-CCL19 and GPC3 CAR-T Cells in the Treatment of Hepatocellular Carcinoma

**DOI:** 10.1155/2021/1782728

**Published:** 2021-09-03

**Authors:** Min Meng, Yi-chen Wu

**Affiliations:** Department of Emergency, The Affiliated Huaian No.1 People's Hospital of Nanjing Medical University, Huaian, Jiangsu, China

## Abstract

**Background:**

Chimeric antigen receptor-modified T cell (CAR-T) therapy has great potential for treating malignant tumors, especially hematological malignancies. However, the therapeutic effect of solid tumors is limited. One of the most important factors is the homing of CAR-T cells to tumor tissues *in vivo*.

**Method:**

a recombinant adeno-associated virus 2 (AAV2) subtype carrying the *CCL19* gene was used to pretreat the tumor before the Glypican-3 (GPC3) CAR-T treatment. The tumor tissue continuously expressed CCL19 and analyzed the tumor-suppressive effect of AAV-CCL19 on GPC3 CAR-T by in vitro and in vivo experiments.

**Result:**

Under the chemotaxis of CCL19, CAR-T cells had a significant increase in the degree of tumor tissue infiltration; also, the antitumor effect *in vitro* was significantly enhanced. AAV-CCL19 combined with GPC3 CAR-T significantly increased the survival time of mice. The aforementioned results showed that the combination of AAV-CCL19 and GPC3 CAR-T cells effectively increased the ability of CAR-T cells to go home into the tumor tissue, making the CAR-T cell treatment more effective.

**Conclusion:**

This study is expected to solve the dilemma in treating CAR-T cell solid tumors and achieve better clinical results.

## 1. Introduction

Human hepatocellular carcinoma (HCC) is the fifth leading cause of cancer deaths [1]. It is one of the most prevalent malignancies in China [2]. The current clinical treatment options are relatively limited for HCC. Surgery is still the best treatment for HCC.

Immunotherapy has been gradually applied to the treatment of malignant HCC in recent years [2, 3]. In China, carrizumab has been approved for treating patients with advanced HCC who have been treated with sorafenib and/or oxaliplatin-containing chemotherapy. The chimeric antigen receptor-modified T cell therapy has been used for treating malignant HCC as an immunotherapeutic agent, serving as a breakthrough in the treatment of hematologic malignancies [4–6]. The phase I clinical data published by Shi et al. on CAR-T cell-targeting GPC3 protein for treating HCC have shown the safety of CAR-GPC3 T cell therapy [7]. However, the therapeutic efficacy was inferior to that of CD19 CAR-T cells for B-ALL. One of the main reasons was that CAR-T cells did not properly go home to tumor tissues, especially memory CAR-T cells with efficient amplification ability [8–10].

Adachi et al. clarified that CCL19 effectively recruited T cells and DC cells to infiltrate tumor tissues [11]. However, the coexpression site of CCL19 and CAR depends on the tropism of CAR-T cells *in vivo*. Memory T cells are more likely to go home to lymph node tissues through the action of proteins such as CCR7 and CD62L [12]. Therefore, generating effective CCL19 in tumor tissues may be one of the important factors to address lymph node homing of CAR-T cells *in vivo*.

Therefore, a subtype 2 adeno-associated viral vector overexpressing CCL19 was constructed. The expression of CCL19 within the tumor could be effectively promoted by intratumor injection of AAV-CCL19. *In vivo* studies showed that CCL19 overexpression within the tumor could effectively recruit CAR-T cells targeting GPC3, an HCC-specific antigen, to infiltrate tumor tissue and better inhibit tumor tissue growth.

## 2. Results

### 2.1. Preparation of CAR-T Cells and AAV-CCL19 Viruses

**Figure 1:**
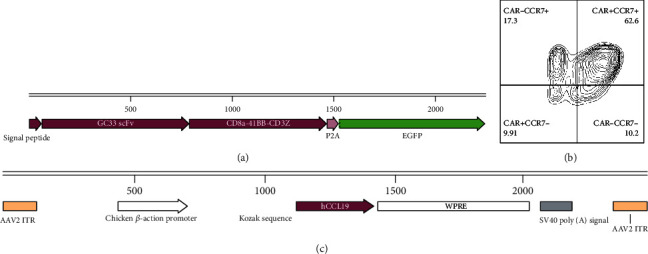
CAR-T and AAV vector characteristics. (a) Schematic diagram of the structure of CAR gene; (b) flow analysis of the CAR-T cell phenotype; (c) schematic diagram of the AAV-CCL19 vector structure.

The structure of CAR protein is shown in Figure 1(a). The single-chain antibody targeting GPC3 fused with the hinge and transmembrane regions of CD8a constitutes the extracellular antigen recognition region; the intracellular signal regions are 4-1BB and CD3z. The GFP was coexpressed with CAR linked by a 2A linker. After the preparation of CAR-T cells, the CAR-T cell memory phenotype was tested. As shown in Figure 1(b), CCR7+ CAR-T cells accounted for about 63%.

As the AAV-2 subtype has a broad spectrum of infection ability *in vivo*, the AAV-2 subtype was chosen in this study. The AAV structure design is shown in Figure 1(c). The cDNA of CCL19 was cloned into the AAV vector downstream CAG promoter. After completing the virus packaging and concentration, the virus titer was tested by qPCR.

### 2.2. Chemotactic Capacity of Overexpressed CCL19 toward Memory CAR-T Cells *In Vitro*

**Figure 2:**
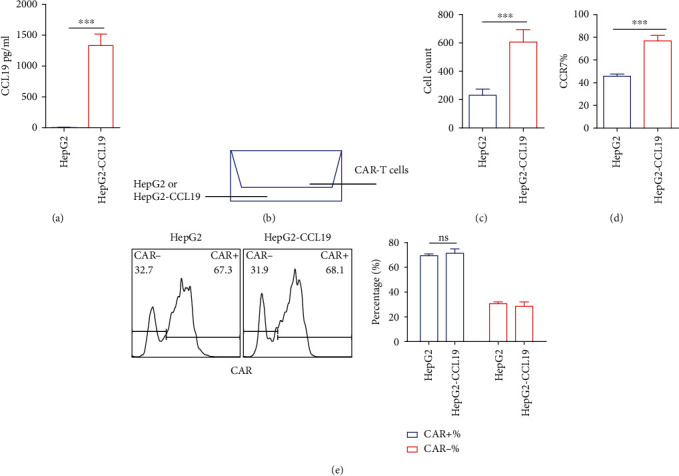
In vitro chemotaxis of CAR-T by CCL19. (a) Detection of CCL19 expression levels of HepG2 and HepG2-CCL19 by ELISA; (b) schematic diagram of the principle of chemotaxis experiment; (c) analysis of HepG2 and HepG2-CCL19 on CAR-T chemotaxis; (d) cell phenotype; (e) positive rate of CAR. Error bars represent mean ± SD (*n* = 3); ns: no significant difference ^∗∗^*P* < 0.01; ^∗∗∗^*P* < 0.001.

Previous studies have shown that CCL19 produced by fibroblast reticular cells in the T zone was essential for the formation and maintenance of the T cell zone in lymphoid organs [13]. T cells are attracted to lymph nodes through the recruitment of CCL19. Therefore, the study first verified whether overexpression of CCL19 by virus-infected tumor cells could recruit CAR-T cells. Lentiviral expression instead of AAV was used to infect HepG2 tumor cells due to the poor infection ability of AAV *in vitro*. After confirming using ELISA that the infected HepG2 cells expressed CCL19 (HepG2-CCL19) (Figure 2(a)), this study verified chemotaxis using the transwell method. Briefly, the HepG2 or HepG2-CCL19 cells were seeded in the lower chamber and the CAR-T cells were seeded in the upper chamber. After 4 h, the cells in the lower chamber were collected and stained using CCR7 and CD3 antibodies, analyzed, and counted by flow cytometry (Figure 2(b)). As shown in Figures 2(c) and 2(d), the number of T cells was higher in the HepG2-CCL19 group than in the HepG2 group. Moreover, the CAR-positive T cells and CAR-negative T cells had the equivalent ability to undergo chemotaxis. The results indicated that CCL19 expressed by tumor cells had a chemotactic effect on CCR7-positive T cells.

### 2.3. Detection of AAV-CCL19 Expression Efficiency *In Vivo*

**Figure 3:**
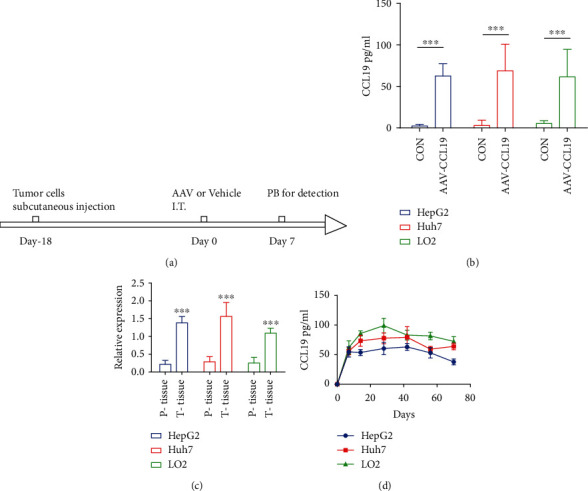
AAV-CCL19-mediated CCL19 expression in vivo. (a) Schematic diagram of in vivo experimental schedule; (b) detection of the CCL19 expression level in peripheral blood 7 days after intratumoral injection of AAV-CCL19; (c) analysis of CCL19 transcript levels in tumor and paraneoplastic tissues by qPCR; (d) long-term detection of CCL19 expression levels in peripheral blood. Error bars represent mean ± SD (*n* = 5); ns: no significant difference ^∗∗∗^*P* < 0.001.

Three cell line-derived xenograft (CDX) models of liver cancer were constructed, HepG2, Huh7, and LO2, to confirm whether AAV-CCL19 injected into tumors could effectively infect tumor cells and express CCL19. The subcutaneous tumors were formed for about 18 days, and 1 × 10^10^ virus particles were injected into each mouse tumor. After 7 days, the peripheral blood of the mouse was taken for CCL19 ELISA (Figure 3(a)). As shown in Figure 3(b), the CCL19 level in the peripheral blood of each mouse injected with AAV-CCL19 increased significantly compared with that in the noninjected group. Mouse tumor tissues and adjacent tissues were taken for RT-qPCR analysis targeting hCCL19 to confirm that CCL19 was expressed by tumor tissues rather than by adjacent cells. As shown in Figure 3(c), although the adjacent tissues had significant expression compared with the control group, the expression level was still significantly lower than that in the tumor tissues. Moreover, the study explored how long AAV-CCL19 could express CCL19 *in vivo*. AAV-CCL19 was injected into the tumor tissues of the three CDX mouse models mentioned earlier by intratumoral injection. The peripheral blood was taken every 7 days to detect the expression of CCL19 in the peripheral blood. The results are shown in Figure 3(d). The three tumor models all had a significant improvement compared with that in the control group. No significant difference was found in the expression level in the three tumor models, and the expression lasted for more than 70 days. The results indicated that AAV-CCL19 effectively infected tumor cells and caused continuous expression of CCL19.

### 2.4. Antitumor Activity of AAV-CCL19 Combined with GPC3 CAR-T Cells *In Vivo*

**Figure 4:**
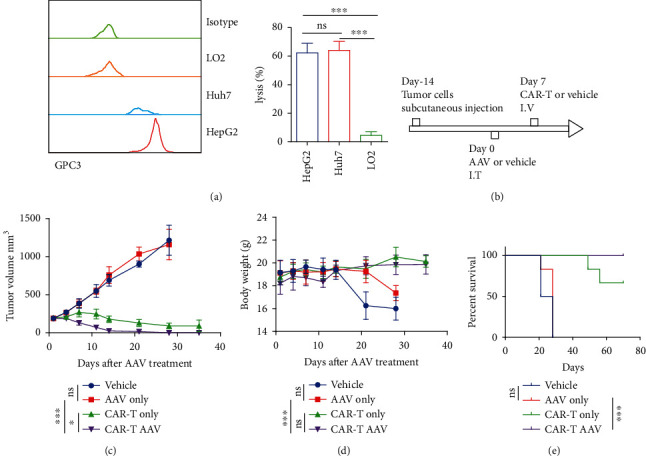
Tumor-suppressive activity of AAV-CCL19 and GPC3 CAR-T. (a) GPC3 expression on the surface of tumor cells detected by FACS and GPC3 CAR-T tumor lysis activity analysis in vitro; (b) schedule of in vivo tumor suppressor activity analysis; (c) in vivo tumor suppression curve; (d) body weight curve; (e) survival curve for mice. Error bars represent mean ± SD (*n* = 5); ns: no significant difference, ^∗^*P* < 0.05, ^∗∗∗^*P* < 0.001.

This study verified the cytotoxicity of GPC3 CAR-T cells against three HCC tumor cells *in vitro*. The results are shown in Figure 4(a). GPC3 CAR-T cells had an obvious killing effect on GPC3-positive tumor cell lines HepG2 and Huh7, while GPC3-negative tumor cell lines had no killing activity (Figure 4(a)). Next, the study verified whether the combination of CAR-T cells and AAV-CCL19 exhibited better tumor-suppressive activity compared with CAR-T and AAV-CCL19 alone. The schedule is shown in Figure 4(b). AAV-CCL19 or phosphate-buffered saline (PBS) was injected into the tumor 14 days after tumor formation, and GPC3 CAR-T cells or PBS was injected into the tail vein on the following 7 days. The body weight and tumor volume were measured once or twice a week. The results are shown in Figures 4(c) and 4(d). The combination group of GPC3 CAR-T cells and AAV-CCL19 (CAR − T + AAV) showed the best antitumor effect. In addition, the AAV-CCL19 single-use group did not exhibit obvious tumor suppressor activity compared with the control group, indicating that AAV-CCL19 had no *in vivo* cytotoxicity. Neither the CAR-T group nor the CAR-T AAV group had a significant effect on the weight of the mice, while the control group and the AAV group were affected by the increase in tumor volume; also, the weight was significantly reduced. The survival time of mice was significantly prolonged in the CAR-T AAV group compared with the control and AAV groups (Figure 4(e)).

### 2.5. AAV-CCL19 Increased the Ability of CAR-T Cells to Chemoattract Tumor Tissues

**Figure 5:**
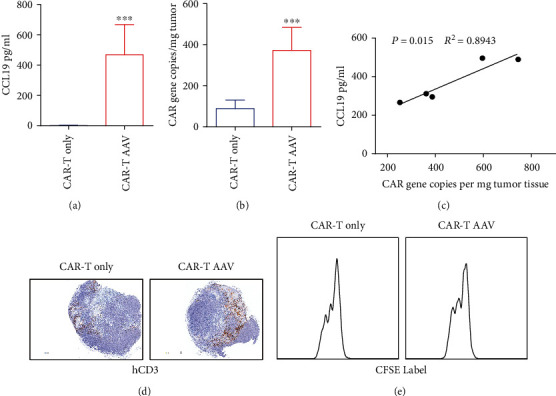
Chemotaxis and infiltration of CAR-T cells by intratumoral injection of AAV-CCL19. (a) Analysis of CCL19 levels in peripheral blood; (b) analysis of the CAR gene content in tumor tissues; (c) Correlation analysis between infiltrated CAR-T cells and CC19 concentration in peripheral blood; (d) human CD3 T cell data performed by IHC after 7 days CAR-T infusion; (e) analysis of proliferation of infiltrated T cell performed by FACS. Error bars represent mean ± SD (*n* = 5); ns: no significant difference ^∗∗^*P* < 0.01; ^∗∗∗^*P* < 0.001.

GPC3 CAR-T cells were injected via the tail vein into tumor-bearing mice treated with or without AAV-CCL19 for 7 days to further verify whether the better antitumor effect produced by the combination of CAR-T cells and AAV was due to the chemotactic effect of CCL19 on T cells. The tumor tissues were removed after 7 days. After grinding, the CCL19 level in the supernatant was detected by ELISA. After cell lysis, quantitative PCR of the CAR gene was performed (Figure 5(a)). The results showed that the expression of CCL19 was significantly higher in the CAR-T AAV group than in the CAR-T group. In addition, the CAR gene copy number was also significantly higher in the CAR-T AAV group than in the CAR-T group (Figure 5(b)). The correlation between the CAR copy number and the CCL19 content was analyzed for the six mice in the CAR-T AAV group. The results are shown in Figure 5(c); the CCL19 content positively correlated with the CAR gene copy number. For immunohistochemistry, the human CD3 (hCD3) was stained and the result was shown in Figure 5(d). The count of infiltrated hCD3 cells in the CAR-T AAV group is more than that in the CAR-T only group. However, the time of division is similar as shown in Figure 5(e). The results suggested that the AAV-mediated expression of CCL19 in tumor cells promoted the infiltration of CAR-T cells into tumor tissues and improved the antitumor activity of CAR-T cell therapy.

## 3. Discussion

In this study, an AAV virus-expressing CCL19 and CAR-T cells targeting the GPC3 antigen were innovatively combined for treating HCC *in vitro* and *in vivo*. *In vitro* and *in vivo* studies have shown that CCL19 expression mediated by AAV-CCL19 inside tumor tissues could promote the migration of memory T cells, including memory CAR-T cells, to the inside of tumor tissues and increase the number of CAR-T cells infiltrating the inside of tumors to achieve better tumor suppression.

The CAR-T therapy has shown encouraging therapeutic results for malignant hematologic diseases such as B-cell lineage leukemia and lymphoma [14]. However, the same therapeutic efficacy has not been achieved for treating solid tumors [7, 8]. Insufficient infiltration capacity, especially of memory CAR-T cells, is one of the main reasons for the unsatisfactory therapeutic results. CCR7, the ligand of CCL19, is expressed on the surface of memory T cells as well as antigen-presenting cells and acts as a homing receptor to promote cell homing to lymph nodes [12, 15]. The activation of T lymphocytes is facilitated by the homing of antigen-presenting cells and memory T cells to lymph nodes. However, memory CAR-T cell activation is dependent on CAR signaling, not TCR signaling. Therefore, memory CAR-T cells cannot be fully activated in the lymph nodes. Studies have shown that memory CAR-T cells are extremely important in treating tumors with CAR-T therapies targeting CD19 [16, 17]. This can be explained by the fact that memory CAR-T cells tend to divide and proliferate when activated by tumor cells. For treating HCC, it is crucial to infiltrate the memory CAR-T cells into the tumor tissue [9]. In this study, the therapeutic effect was increased by the intratumoral injection of AAV-CCL19 to make tumor cells express CCL19 and recruit and promote memory CAR-T cells to infiltrate into the tumor interior.

Type 2 recombinant adeno-associated virus is a nonintegrated, genomic, nonreplicating virus vector. AAV-2 is widely available in host cells *in vivo*, with high expression and low immunogenicity [18, 19]. Therefore, it is widely used for treating many diseases. On the contrary, no relevant attempts have been made for solid tumor treatment. In this study, considering safety issues, AAV was used as a vector to express CCL19 and recruit CAR-T cells. Intratumorally injected AAV-CCL19 had no toxic effects on paraneoplastic tissues despite their expression on paraneoplastic cells (probably due to leakage during injection). Keishi et al. reported that the coexpression of CCL19 and IL-7 with CD20-targeted CAR-T cell therapy could effectively increase CAR-T cell and T cell infiltration [11]. However, CAR-T cell distribution convergence *in vivo* was not obvious. Despite intratumoral injection, CAR-T cells could still migrate into extratumoral tissues, affecting CAR-T cell tropism mediated by the overexpression of CCL19. In the present study, AAV-CCL19 was injected intratumorally to recruit CAR-T cells. Since rAAV cannot replicate *in vivo* and therefore cannot migrate to other locations after infecting tumor cells, the CCL19 expression mediated by AAV-CCL19 might be more chemotactic compared with that mediated by CAR-T.

In summary, the combination of AAV-CC19 and GPC3-targeted CAR-T cell therapy designed in this study effectively increased the migration and infiltration of CAR-T cells into tumor tissues and increased the therapeutic effect of CAR-T cells. This study provided a new therapeutic idea for treating HCC.

## 4. Method

### 4.1. Construction of Plasmid Vectors

The human CCL19 gene cDNA was subjected to full gene synthesis to construct the AAV-CCL19 plasmid vector. The synthesized gene was cloned between the *Not*I and *Bam*HI digestion sites of the pAAV.CMV.PI.EGFP.WPRE.bGH plasmid (obtained from Addgene; plasmid #105530). CAR genes were synthesized as described previously to construct a lentiviral vector expressing GPC3 CAR. Briefly, the genes related to GC33 scFv, CD8a hinge, and transmembrane regions and CD137 and CD3zeta intracellular regions were sequentially ligated, fully genetically synthesized, and cloned between the *Eco*RI and *Mlu*I digestion sites of plasmid pLVX-EF1a-PTS1-IRES-Puromycin (obtained from Addgene; plasmid #134665).

### 4.2. Packaging of AAV and Lentivirus

AAV-CCL19 virus was packaged as described in a previous study. Briefly, the AAV-CCL19 core plasmid and two helper plasmids were cotransfected into 293T cells. The cells were harvested 48 h later and purified according to the AAVpro Purification Kit (TaKaRa; 6232) instructions. After virus purification, virus titers were measured using the AAVpro Titration Kit (for real-time PCR) Ver. 2 (TaKaRa, 6233). For lentivirus packaging, the constructed core plasmid and two helper plasmids were cotransfected into 293 cells. The supernatant was recovered after 48 h, and the lentivirus was concentrated using the Lenti-X Concentrator (TaKaRa, 631231). The concentrated lentivirus was titered with Lenti-X GoStix Plus (TaKaRa, 631280).

### 4.3. CAR-T Cell Preparation

PBMCs isolated from healthy volunteers were used to isolate T cells with EasySep Human CD3 Positive Selection Kit (STEMCELL, 18051). The T cells were activated with cured OKT3 and OK28 antibodies and Retronectin (TaKaRa, T100B). After 24 h, the lentivirus (MOI = 1) was added and incubated for 24 h. Subsequent T cell densities were maintained at 1 million to 2 million T cells per mL. A T cell culture medium formulation was as follows: X-VIVO 15 serum-free hematopoietic cell medium containing 100 IU/mL IL-2 and penicillin–streptomycin.

### 4.4. Flow Cytometry

The cells were stained with flow-through antibodies by centrifugation at 200 g for 5 min at room temperature, and the supernatant was removed. They were resuspended in 100 *μ*L of PBS, and 1 *μ*g of the antibody was added. They were incubated for 30 min at 4°C, washed twice with PBS, resuspended in 200 *μ*L of PBS, and detected by flow cytometry.

### 4.5. ELISA

The peripheral blood serum or cell supernatant was collected and processed according to the instructions on the Human CCL19/MIP-3 beta DuoSet ELISA kit. The results were read, and the concentration values were calculated using BioTek Synergy Neo2.

### 4.6. Xenogenic Mouse Models

Three million Huh7 cells or five million HepG2 cells or three million LO2 cells were injected subcutaneously into the axilla of 8-week-old NOG mice. Tumor volumes were measured twice weekly using Vernier calipers. AAV-CCL19 was injected intratumorally after 18 days, and the peripheral blood was taken for ELISA 7 days after the injection. CAR-T cells were injected into the mice by tail vein injection. The tumor volume and mouse body weight were checked twice a week.

### 4.7. Statistical Analyses

GraphPad Prism 6.0 was used for statistical analysis. One-way or two-way analysis of variance with Bonferroni post test or unpaired, two-tailed *t*-tests were used as appropriate. Symbols indicated statistical significance (^∗^*P* < 0.05; ^∗∗^*P* < 0.01; ^∗∗∗^*P* < 0.001). Each experiment was performed at least three times.

## Data Availability

The data used to support the findings of this study are available from the corresponding author upon request.
